# Association of Supporting Trial Evidence and Reimbursement for Off-Label Use of Cancer Drugs

**DOI:** 10.1001/jamanetworkopen.2021.0380

**Published:** 2021-03-02

**Authors:** Amanda Katherina Herbrand, Andreas M. Schmitt, Matthias Briel, Hannah Ewald, Marius Goldkuhle, Stefan Diem, Anouk Hoogkamer, Markus Joerger, Giusi Moffa, Urban Novak, Lars G. Hemkens, Benjamin Kasenda

**Affiliations:** 1 Department of Medical Oncology, University Hospital Basel and University of Basel, Basel, Switzerland; 2 Department of Internal Medicine, St Claraspital, Basel, Switzerland; 3 Basel Institute for Clinical Epidemiology and Biostatistics, Department of Clinical Research, University Hospital Basel and University of Basel, Basel, Switzerland; 4 Department of Health Research Methods, Evidence, and Impact, McMaster University, Hamilton, Canada; 5 University Medical Library, University of Basel, Basel, Switzerland; 6 Department of Internal Medicine, University of Cologne, Center for Integrated Oncology Aachen Bonn Cologne Duesseldorf, Cologne, Germany; 7 Department of Oncology and Hematology, Cantonal Hospital St Gallen, St Gallen, Switzerland; 8 Department of Oncology and Hematology, Spital Grabs, Grabs, Switzerland; 9 Department of Mathematics and Computer Science, University of Basel, Basel, Switzerland; 10 Department of Medical Oncology, Bern University Hospital, Bern, Switzerland; 11 Meta-Research Innovation Center Berlin (METRICS-B), Berlin Institute of Health, Berlin, Germany; 12 Meta-Research Innovation Center at Stanford (METRICS), Stanford University, Stanford, California; 13 Research and Development, iOMEDICO, Freiburg, Germany

## Abstract

**Importance:**

In many health systems, access to off-label drug use is controlled through reimbursement restrictions by health insurers, especially for expensive cancer drugs.

**Objective:**

To determine whether evidence from randomized clinical trials is associated with reimbursement decisions for requested off-label use of anticancer drugs in the Swiss health system.

**Design, Setting, and Participants:**

This cross-sectional study used reimbursement requests from routinely collected health records of 5809 patients with drug treatment for cancer between January 2015 and July 2018 in 3 major cancer centers, covering cancer care of approximately 5% of the Swiss population, to identify off-label drug use. For each off-label use indication with 3 or more requests, randomized clinical trial evidence on treatment benefits was systematically identified for overall survival (OS) or progression-free survival (PFS). Data were analyzed from August 2018 to December 2020.

**Exposures:**

Available randomized clinical trial evidence on benefits for OS or PFS for requested off-label use indications.

**Main Outcomes and Measures:**

The main outcome was the association between evidence for treatment benefit (expressed as improved OS or PFS) and reimbursement in multivariable regression models.

**Results:**

Among 3046 patients with cancer, 695 off-label use reimbursement requests in 303 different indications were made for 598 patients (median [interquartile range] age, 64 [53-73] years; 420 [60%] men). Off-label use was intended as first-line treatment in 311 requests (45%). Reimbursement was accepted in 446 requests (64%). For 71 indications, including 431 requests for 376 patients, there were 3 or more requests. Of these, 246 requests (57%) had no supporting evidence for OS or PFS benefit. Reimbursement was granted in 162 of 246 requests without supporting evidence (66%). Of 117 requests supported by OS benefit, 79 (67%) were reimbursed, and of 68 requests supported by PFS benefit alone, 54 (79%) were reimbursed. Evidence of OS benefit from randomized clinical trials was not associated with a higher chance of reimbursement (odds ratio, 0.76, 95% CI, 0.45-1.27).

**Conclusions and Relevance:**

These findings suggest that in a health care system enabling access to off-label use, it was frequently intended as a first-line treatment in cancer care. Availability of randomized clinical trial evidence showing survival benefit was not associated with reimbursement decisions for off-label anticancer drug treatment in Switzerland. A transparent process with criteria considering clinical evidence is needed for evidence-based reimbursement decisions to ensure fair access to cancer treatments.

## Key Points

**Question:**

Is evidence from randomized clinical trials associated with the reimbursement decisions that determine access to off-label use of cancer drugs in Switzerland?

**Findings:**

In this cross-sectional study including 3046 patients with cancer, there were a total of 695 off-label use requests for 598 patients (20%). A total of 67% of requests were reimbursed when supporting trials showed overall survival benefit, but 65% of requests were reimbursed without evidence for overall survival or progression-free survival benefit.

**Meaning:**

These findings suggest that reimbursement for off-label cancer treatment in Switzerland was not evidence-based and that a transparent process considering clinical evidence is urgently needed for reimbursement decisions to guarantee fair access to off-label cancer drugs.

## Teaser

This cross-sectional study examines the association of evidence for benefit from randomized clinical trials with off-label cancer drug reimbursement decisions in Switzerland.

## Introduction

Off-label use refers to the use of drugs outside their indication approved by regulatory agencies^1^ and is common in oncological care.^2^ An estimated 13% to 71% of adult patients with cancer^2^ receive off-label drugs during the course of their treatment, including approximately 30% of patients in the US^3^ and Switzerland.^4^ When evidence about the benefits of a drug in a new indication emerges, off-label use may allow its early use in routine care before formal approval.^5^ A prominent example is the *ERBB2* (OMIM 164870; formerly *HER2*)-directed antibody, trastuzumab, which is approved for *ERBB2*-positive breast cancer.^6,7^ The clear clinical benefit of trastuzumab, with up to 8 months extended survival and doubling of the overall response in patients with advanced breast cancer, has prompted further investigations in other malignant neoplasms. In 2009, a randomized clinical trial (RCT) showed better survival for patients with advanced *ERBB2*-positive gastric cancer who received trastuzumab in addition to chemotherapy.^8^ It took 1 year until formal approval was granted for this new indication by the US Food and Drug Administration (FDA),^9^ and during this time, patients with *ERBB2*-positive advanced gastric cancer could only receive trastuzumab via off-label use.

Cancer drugs are often expensive, and their reimbursement is an economic challenge for health care systems; therefore, off-label use is frequently regulated by reimbursement restrictions in many countries.^10^ In Switzerland, reimbursement of drugs in approved indications is regulated by the Swiss government (based on the approval decisions of Swissmedic, the equivalent to the FDA) and compulsory for all Swiss health insurers. Reimbursement of off-label use is individually determined by Swiss health insurers. The treating physician has to issue an upfront reimbursement request, and the patient’s health insurer decides on a case-by-case basis. Off-label use has to be reimbursed if it is indicated for a life-threatening disease, a substantial benefit can be expected, and no other effective and approved treatment is available.^11^ However, there are no fixed definitions of these criteria, and there is less than full transparency on how Swiss insurance companies appraise whether substantial benefit can be expected. In the US, insurance companies rely on different compendia (eg, American Hospital Formulary Service Drug Information, the US Pharmacopeia Drug Information, or Drugdex) for off-label use reimbursement, regardless of evidence or comprehensiveness. However, legislation for off-label use reimbursement differs from state to state, which carries the risk of inequal access to off-label use treatment.^1,12^

In an evidence-based health care system, the main driving factor for reimbursement decisions should be the available evidence. In this cross-sectional study, we report results a large-scale empirical investigation about the reimbursement reality of off-label use in cancer care in Switzerland, with the aim of understanding the association of supporting evidence with the reimbursement decisions that determine access to off-label use for patients with cancer.

## Methods

This cross-sectional study was approved by the local research ethics committee of North-West Switzerland in Basel. We were granted a waiver of written informed consent by the research ethics committee because this study only included information extracted from routinely collected health care data. This study is part of the Comparative Effectiveness of Innovative Treatments in Cancer—Off Label Use project, described elsewhere.^13^ In brief, we used routinely collected health data from medical care records to investigate the prevalence of intended off-label use in patients with cancer, reimbursement of off-label use, and the association of RCT evidence with reimbursement of off-label use. The reporting follows the Strengthening the Reporting of Observational Studies in Epidemiology (STROBE) reporting guideline.

### Selection and Eligibility Criteria

We screened medical records of patients with a first consultation at 1 of 3 major Swiss oncology and hematology centers (University Hospital Basel, Bern University Hospital, and Cantonal Hospital St Gallen) between January 2015 and July 2018. We included patients with a malignant neoplasm who received at least 1 anticancer drug treatment. From 1 center (University Hospital Basel), we included all eligible patients, and we randomly selected 1000 patients from all eligible patients in each of the other 2 centers.

### Definition of Off-Label Use and Indications

We retrieved approval information of all anticancer drugs from the Swiss drug regulation agency, Swissmedic,^14^ and publicly available repositories.^15^ We considered all requested drug treatments as off-label use if there was at least 1 deviation from the Swissmedic drug label regarding the disease, treatment setting, line of treatment, and application of the drug (eAppendix in the Supplement). All requested drug treatments that were not approved by Swissmedic (also known as *compassionate* or *unlicensed use*^2^) were considered off-label use.

### Evidence From RCTs

For each off-label use indication with at least 3 requests, we systematically determined the evidence supporting an intended off-label use at the time of request (details of the systematic search have been published elsewhere^13^). Two reviewers (A.K.H. and A.M.S.) independently used a prespecified search strategy for PubMed and Google Scholar to identify RCTs testing the drug in the intended off-label indication. We focused on RCT evidence to avoid the high uncertainty associated with benefit assessments based on nonrandomized trials, although we did take into consideration that some drugs are approved based on nonrandomized trials. One independent investigator (M.G.) verified the eligibility of all identified RCTs. We extracted bibliographic details, earliest date of publication (online or print), comparator drug or regimen, patients enrolled, and treatment effects (hazard ratios [HRs] with 95% CIs and *P* values) for overall survival (OS) and progression-free survival (PFS) from each RCT. Whenever possible, we chose the reported effect estimate labeled as intention-to-treat analysis. If no 95% CI was reported but a *P* value was, we used the *P* value to calculate the 95% CI.^16^ For 3 indications, we considered the effect reported from subgroup analyses in RCTs that more closely matched the respective off-label use indication. Finally, for each off-label use reimbursement request, the corresponding evidence available at that specific time was determined (independently by A.K.H. and A.M.S.; verified by B.K.). When there were multiple corresponding RCTs available at that time, their results on OS and PFS were summarized in random-effects meta-analyses. If RCT results were updated during the study period (ie, January 2015 to July 2018), the updated analyses were considered for the respective times. We used 3 categories to describe the available evidence for treatment benefits at the time of each off-label use request: OS benefit (summary HR for OS <1 and upper 95% CI limit <1); PFS benefit (no OS benefit but summary HR for PFS <1 and upper 95% CI limit <1); or no benefit (no OS or PFS benefit or no RCT evidence available at all).

### Statistical Analysis

Our main outcome was the final decision on the reimbursement request (acceptance vs rejection) by the health insurers. The unit of analysis was the individual reimbursement request. We calculated frequencies and proportions of accepted and rejected off-label use requests for each indication and each of the 3 evidence categories. To investigate whether insurance companies were more likely to approve off-label use requests in the presence of evidence for OS or PFS benefit, we used multilevel logistic regression analyses with a 3-level model for our main analyses: the unit of analysis was the reimbursement request and clustering was analyzed by off-label use requests by patient (there can be >1 request per patient) and by patients’ health insurers (decision-making might differ across health insurers). We used 2 main models with different definitions of the exposure variable, which was the level of treatment benefit linked to the individual off-label use request. The first model, OS benefit vs all other categories, including PFS benefit and no benefit, addressed the question of whether patients were more likely to receive acceptance for off-label use when OS benefit had been demonstrated. The second model, OS benefit or PFS benefit vs no benefit, addressed the question of whether patients were more likely to receive acceptance for off-label use when at least PFS benefit had been demonstrated.

The probability of acceptance was expressed as an odds ratio (OR), with an OR greater than 1 indicating a higher chance of acceptance in the presence of evidence for OS or PFS benefit. We created forest plots with crude ORs for every health insurer to visualize the dispersion of reimbursement decisions across health insurances.

#### Association of Reimbursement With Other Factors

To investigate other factors potentially associated with reimbursement decisions, we conducted multilevel multivariable logistic regression analyses including prespecified patient, disease, and treatment characteristics (ie, sex, age, requirement of markers associated with response to the drug, disease type, and request for an immune checkpoint inhibitor (ICI). We did not include 2 other prespecified factors. The first was orphan drug status, as this status is granted during the approval process for a specific drug within a specific indication; therefore, within the Swiss health care system, orphan drug status only applies to drugs given on-label. The second was cancer incidence because we could not reliably identify the required information for many specific disease entities (eg, incidence was available for lung cancer, but not for non–small cell adenocarcinoma of the lung). Ad hoc, we added the line of treatment to the multivariable model (first, second, or third line and beyond) to investigate whether reimbursement requests were more likely to be accepted for patients who had undergone previous lines of treatment.

#### Sensitivity Analysis and Missing Data

To investigate whether clustering had any impact on our estimates, we modified the 2 main models considering clustering by health insurer or patient only and no clustering at all. In a further sensitivity analysis, we only included requests for indications for which an RCT was available (trial model). We anticipated missing data^13^ for our main outcome, and we prespecified analyses based on multiple imputations.

#### Data Processing and Software

We built a relational database (Ninox) to store extracted pseudonymized information from patient records and information from our literature searches. An encryption process using a unique patient identifier was embedded to secure patient privacy. We used R statistical software version 3.5.1 (R Project for Statistical Computing) for all statistical analyses. For meta-analyses and cumulative meta-analyses, we used the R package meta, version 4.9-4 to calculate ORs and forest plots. For the multilevel logistic regression model and sensitivity analyses we used the R package lme4, version 1.1-20. We performed multiple imputations and logistic regression analyses on imputed data sets using Stata version 15.1 (StataCorp). All calculations and analyses were performed in duplicate. Data were analyzed from August 2018 to December 2020.

## Results

### Prevalence of Off-Label Use Requests

We screened 5809 patient records and included 3046 eligible patients (eFigure 1 in the Supplement). At least 1 off-label use reimbursement request was issued for 568 patients (19%), with a total of 695 off-label use requests. Reimbursement was accepted in 446 requests (64%) and rejected in 185 requests (27%). Decisions on reimbursement were missing for 64 requests (9%). Overall, we identified 303 different off-label use indications (Table 1; eTable 1 in the Supplement).

**Table 1.** Top 10 Most Frequently Requested Off-Label Use Indications Between January 2015 and July 2018^a^

Indication	Treatment line	Drug	Overall requests, No.	Decision, No. (%)	
				Accepted	Not available
AML or MDS, postallogenic SCT, single agent	Any, but mostly first	Azacitidine	31	22 (71)	0
Breast cancer, adjuvant, drug combination	First	Zoledronic acid	28	21 (75)	1 (4)
Multiple myeloma, postautologous SCT, single agent	First	Lenalidomide	26	20 (77)	6 (23)
Melanoma, adjuvant, single agent	First	Nivolumab	16	16 (100)	0
Prostate cancer, advanced, drug combination	First	Abiraterone	14	11 (79)	1 (7)
Glioblastoma, advanced, single agent	Second, third, or beyond	Lomustin	13	9 (69)	3 (23)
Follicular lymphoma, induction, single agent	First	Rituximab	11	11 (100)	0
Multiple myeloma, induction, drug combination	First	Bortezomib	9	8 (89)	1 (11)
Pancreatic cancer, adjuvant, drug combination	First	Capecitabine	9	7 (78)	1 (11)
SCLC, advanced, drug combination	Second, third, or beyond	Ipilimumab	9	4 (44)	0
Pancreatic cancer, advanced, or beyond, drug combination	Second, third, or beyond	Nab Paclitaxel	9	6 (67)	0
NSCLC, adenocarcinoma, advanced, drug combination	First	Pembrolizumab	9	7 (78)	0
Posttransplant lymphoproliferative disorder, induction, single agent	First	Rituximab	9	7 (78)	2 (22)
AML, postallogenic SCT, single agent	First, second, third, or beyond	Sorafenib	9	5 (56)	1 (11)

Abbreviations: AML, acute myeloid leukemia; MDS, myelodysplastic syndrome; NSCLC, non–small cell lung carcinoma; SCLC, small cell carcinoma; SCT, stem cell transplantation.

^a^ There are more than 10 indications listed because different indications can have the same number of requests.

### Characteristics of Off-Label Use Requests

Among 695 requests, the median (interquartile range) patient age was 64 (53-73) years, 420 requests (60%) were for male patients, and 441 requests (63%) were to treat a solid tumor (Table 2). A total of 133 requests (19%) were for ICIs, and evidence of a biomarker associated with treatment response was required for 118 requests (17%). Most off-label use requests were intended as first-line (311 requests [45%]) or second-line therapy (215 requests [31%]). Rejections were more common for reimbursement requests for ICIs (136 rejections of 441 requests [31%]) and solid tumors (53 rejections of 133 requests [40%]) compared with requests for non-ICIs (130 rejections of 562 requests [23%]) and nonsolid tumors (47 rejections of 254 requests [19%]). Of the top 14 most frequently requested off-label use indications in our study (Table 1), 11 (79%) were off-label use in the US by January 2015. Four indications (29%) were later approved during the study period in the US and Switzerland.

**Table 2.** Patient, Disease, and Treatment Characteristics of Reimbursement Requests

Characteristic	No. (%)			
	All (n = 695)	Reimbursement decision		
		Accepted (n = 450)	Rejected (n = 183)	Unknown (n = 62)
Patient				
Age, median (IQR), y	64 (53-73)	64 (53-72)	65 (54-75)	64 (55-73)
Men	420 (60)	267 (59)	113 (62)	40 (65)
Tumor type				
Hematological diseases	149 (21)	104 (23)	32 (17)	13 (21)
Lymphoma	105 (15)	78 (17)	15 (8)	12 (19)
Solid tumor	441 (63)	268 (60)	136 (74)	37 (60)
Drug type				
Immune checkpoint inhibitor	133 (19)	76 (17)	53 (29)	4 (6)
Targeted therapy	118 (17)	81 (18)	30 (16)	7 (11)
Line of treatment				
First	311 (45)	222 (50)	65 (36)	24 (39)
Second	215 (31)	129 (29)	66 (36)	20 (33)
≥Third	166 (24)	97 (22)	52 (28)	17 (28)

Abbreviation: IQR, interquartile range.

### Evidence and Reimbursement Reality

For 71 indications, at least 3 different reimbursement requests were issued, covering 431 of 695 off-label use requests (62%) and 376 of 568 patients (66%). The 3 most frequently requested indications were azacitidine for acute myeloid leukemia or myelodysplastic syndrome as maintenance therapy after allogeneic stem cell transplantation (31 requests), zoledronic acid for breast cancer as adjuvant treatment in combination with antihormonal therapy (28 requests); and lenalidomide for multiple myeloma as maintenance therapy after autologous stem cell transplantation (26 requests). Of these 71 indications, 29 indications (41%) were tested in a single RCT, 7 indications (10%) were tested in 2 RCTs, and 4 indications (6%) were tested in 3 RCTs. For 31 indications (44%), we found no RCT evidence.

For 14 of 71 indications (20%), all requests were approved (1 indication with OS benefit; 3 indications with PFS but not OS benefit; 10 indications without OS or PFS benefit). For 5 indications (7%), all requests were denied (1 indication with OS benefit; 1 indication with PFS but no OS benefit; 3 indications without OS or PFS benefit), and for 59 indications (63%) reimbursement decisions were inconsistent, ie, some requests were approved and some were not.

**Figure 1.**
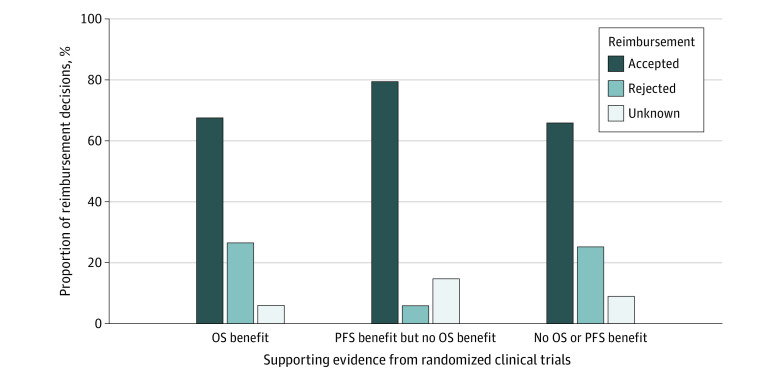
Proportions of Accepted and Rejected Off-Label Reimbursement Requests Per Category of Supporting Evidence From Randomized Clinical Trials OS indicates overall survival; PFS, progression-free survival.

Among 431 requests in these 71 indications, 117 requests (27%) were supported by RCT evidence showing an OS benefit, and 68 requests (16%) were supported by RCT evidence showing a PFS benefit but no OS benefit. The remaining 246 requests (57%) were not supported by RCT evidence for OS or PFS benefit. For 25 of these 246 requests (10%), RCTs did not reveal an OS or PFS benefit, and 221 of these 246 requests (90%) were not supported by RCT evidence investigating OS or PFS at all (eTable 2 in the Supplement). Overall, 162 of 246 requests (66%) were approved without supporting RCT evidence for an OS or PFS benefit, 54 of 68 requests (79%) were approved with evidence for PFS benefit (but not for OS), and 79 of 117 requests (67%) were approved with evidence for an OS benefit (Figure 1).

**Figure 2.**
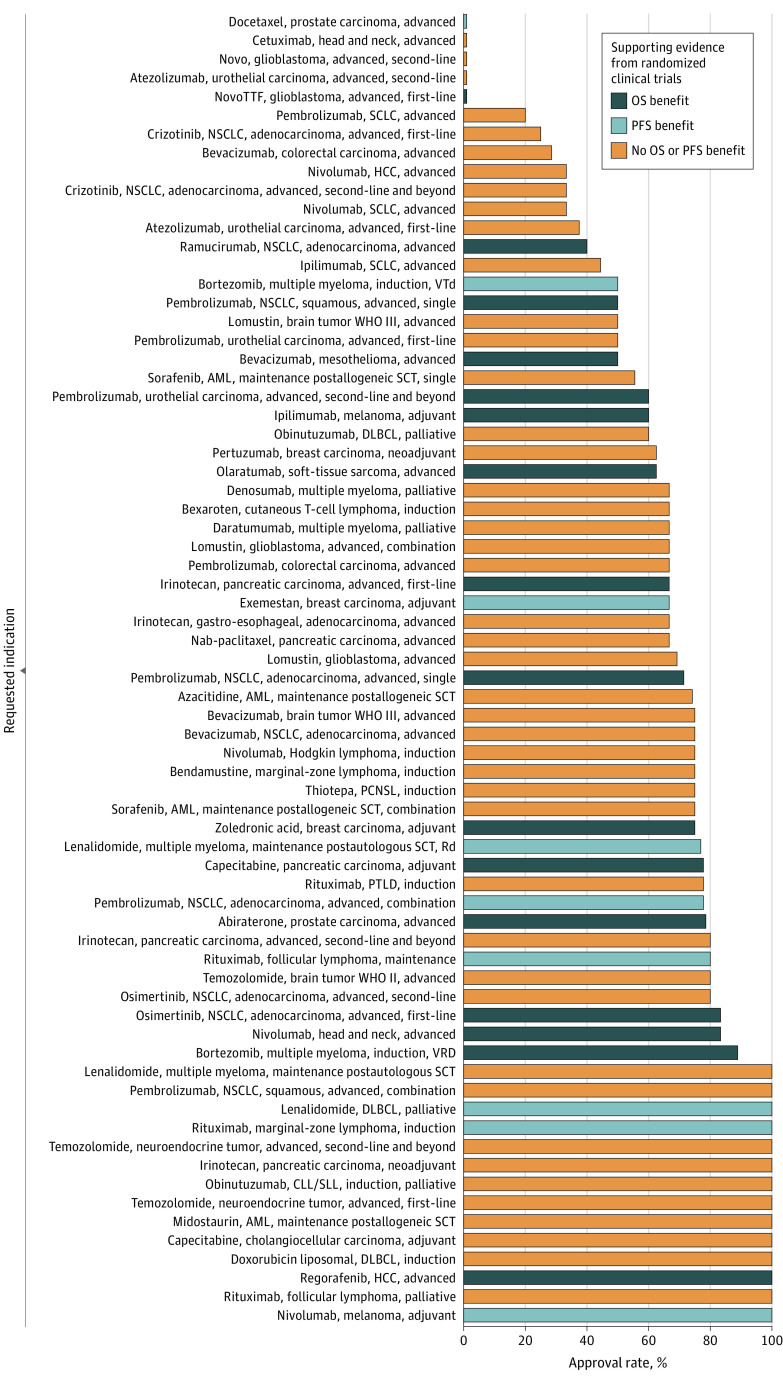
Proportion of Off-Label Reimbursement Approvals for All 71 Indications With at Least 3 Requests in the Study Period AML, acute myeloid leukemia; CLL, chronic lymphocytic leukemia; combination, drug requested in combination with other drug treatment; DLBCL, diffuse large B-cell lymphoma; HCC, hepatocellular carcinoma; NSCLC, non–small cell lung cancer; PCNSL, primary central nervous system lymphoma; PTLD, posttransplant lymphoproliferative disorder; Rd, lenalidomide, dexamethasone; SCLC, small cell lung cancer; SCT, stem cell transplantation; single, monotherapy; SLL, small lymphocytic lymphoma; VRD, bortezomib, lenalidomide, dexamethasone; VTd, bortezomib, thalidomide, dexamethasone; and WHO, World Health Organization grade.

In multilevel regression analysis, the chance of reimbursement was not higher in the presence of RCT evidence for OS benefit compared with all other situations (OR, 0.76; 95% CI, 0.45-1.27) (Figure 2). This was consistent in the sensitivity analysis using a multivariable model (OR, 0.85; 95% CI, 0.47-1.54) (eTable 3 in the Supplement). The multilevel regression analysis did not suggest a higher chance for reimbursement in the presence of RCT evidence for OS or PFS benefit compared with situations without such evidence (OR, 1.46; 95% CI, 0.91-2.36), although the sensitivity analysis using a multivariable model was compatible with an almost 2-fold higher chance of reimbursement (OR, 1.79; 95% CI, 1.01-3.18) (eTable 3 in the Supplement). Reimbursement requests for ICIs and second-line therapies were rejected more frequently (eTable 3 in the Supplement). Results were similar in the other sensitivity analyses (eTable 4 in the Supplement).

**Figure 3.**
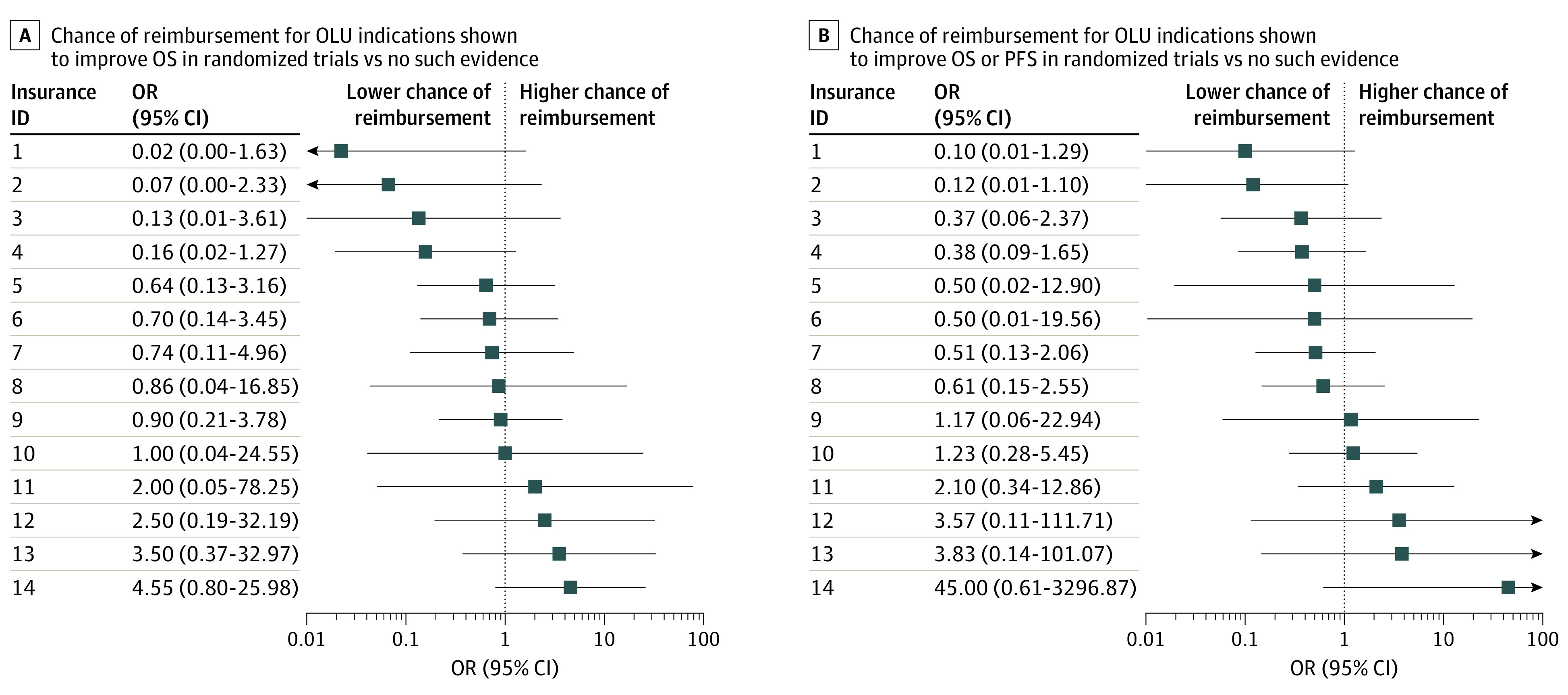
Forest Plot of the Crude Odds Ratios (ORs) for Acceptance of Reimbursement Requests for Off-Label Use (OLU) Across All Indications for Each Health Insurer With at Least 5 Reimbursement Requests An OR greater than 1 indicates a higher chance of acceptance of reimbursement in the presence of supporting evidence. For 2 insurers, no OR was calculated because all reimbursement requests were accepted. Insurers are identified by number for anonymity.

The results stratified by health care insurers suggested no association between available evidence for benefit and positive reimbursement decisions (Figure 3). Even within the same health insurer for the same indication, we found no consistency in decision making (eFigure 2 in the Supplement).

## Discussion

This cross-sectional study of more than 3000 patients with a large variety of cancer types showed that for 1 in 5 patients, off-label use was an intended treatment. While it is widely assumed that off-label use is typically a treatment for rare diseases and in situations of exhausted approved treatment options,^17,18,19^ our findings suggest that off-label use was typically intended as a first- or second-line treatment option. While health insurers provided access to off-label use by accepting reimbursement in 64% of requests, their decisions were not associated with the supporting evidence from RCTs at the time of the request. Approximately 1 in 3 reimbursement requests were rejected, regardless whether there was RCT evidence supporting it. Although there was a positive association of level of evidence with the chance for reimbursement in 1 sensitivity analysis, this observed association was not consistent. Results from nonrandomized trials may be important to justify off-label use in some situations; however, in an evidence-based health care system, intended off-label use supported by the most reliable evidence (ie, from RCTs) showing improved survival would be expected to always have the highest chance to be reimbursed. However, even within the same health insurer, reimbursement decisions on the same drug in the same off-label use indication with the same supporting evidence for benefit were inconsistent. Thus, most patients with cancer in Switzerland had access to off-label use, but the decision-making process by the health insurer was not transparent to the requesting oncologist or hematologist and not evidence-based.

In contrast to previous assumptions, our study found that 45% of off-label use requests were intended for first-line treatment, suggesting that off-label use was often the preferred primary therapy for patients with cancer. This may partly be explained by a time lag between availability of evidence (eg, from peer-reviewed publications) and formal approval of new indications. For example, for FDA approvals, the median (range) lag time has been described as 7 (0.6-13.2) months, and it is more than the median (range) of 12 (4.9-22.4) months for the European Medicines Agency.^20^ Another reason that treatments are considered off-label use, even though they may be already established as a standard of care, is the lack of incentives for pharmaceutical companies to apply for an expensive and sometimes lengthy approval process.^19^

Swiss law mandates reimbursement of requested off-label use in cases of a potentially fatal disease without an effective approved alternative therapy.^11^ However, the law leaves room for interpretation, and decisions on off-label use reimbursement are entirely up to the health insurer. In addition, it is not clear if medical reviewers use the evidence attached to the request by the issuing physician, conduct standardized searches for evidence on their own, or consult guidelines. This is different to the US system,^21^ in which health insurers at least need to consult guidelines developed by 5 different compendiums.^22^ Usually, US health insurers reimburse off-label use if it is endorsed by such a guideline, regardless of the evidence. Of the top 14 most frequently requested off-label use indications in our study, 11 (79%) were also off-label use in the US by January 2015, according to the FDA approval status. Of those 14 indications, 4 were approved during the study period in the US and Switzerland.

A 2018 survey of the evidence underlying off-label use based on the National Comprehensive Cancer Network guidelines^22^ reported that only 23% of the off-label use indications were supported by evidence from RCTs, and a study by Radley et al^23^ reported that outside of oncological and hematological treatments, 73% of all off-label use prescriptions by office-based physicians were supported by little or no scientific evidence. This illustrates that there are situations in which clinicians feel confident to treat patients with off-label use even in the absence of sufficient evidence. Such situations might be justified and driven by convincing results from nonrandomized trials, strong patient preferences, or a sound rationale based on a biomarker associated with treatment response. However, there are examples calling for caution when making premature inferences on treatment benefits in the absence of RCT evidence, even within the same tumor type. For example, irinotecan and bevacizumab are effective and approved treatments for metastatic colorectal cancer,^24,25^ and one might conclude that this would also translate into the adjuvant setting. However, later RCTs could not show any benefit in an adjuvant treatment setting, but they did find a possible detrimental effect on patient survival.^26,27^

Tumor site agnostic treatment approaches, in which not the site of the tumor but common molecular alterations determine treatment choices, will very likely increase off-label use in the future.^28^ In our study, the presence of a biomarker associated with treatment response was prerequisite for the use of 17% of requested off-label use indications. However, drug prices have skyrocketed in the last 30 years, thus increasing off-label use is likely to become a challenge to any health care system, especially if reimbursement is not systematically regulated. Neither the US nor Swiss reimbursement systems currently seem fit to deal with the increased burden that off-label use is likely to impose in the future.

Our results are based on a representative sample with almost 6000 patients covering approximately 5% of all annual patients with cancer in Switzerland.^29^ By extracting data on disease and treatment history and the correspondence with the health insurer, we have established a unique data set for investigating which factors are associated with reimbursement decisions in Swiss cancer care.

### Limitations

This study has several limitations. First, our analysis relies on medical records and the quality of the documentation. In approximately 10% of reimbursement requests, we could not find the final decision on reimbursement in the files, but sensitivity analyses suggested that missing data had no substantial impact on our results. Second, we only searched for RCTs because they provide the most reliable evidence. The finding that reimbursement decisions were inconsistent despite proven OS benefit in RCTs suggests structural problems beyond different interpretations of study designs. Third, we focused on treatment effects on OS and PFS but did not consider quality of life. Quality of life is a rarely explored end point in pivotal RCTs of new cancer drugs,^30^ and benefits on quality of life are not explicitly considered in the legal requirements for reimbursement of off-label use in Switzerland.^11^ Fourth, we did not assess the quality of the included RCTs, which has been described as being often limited.^31^ Fifth, we focused on off-label use indications that had been requested at least 3 times, covering more than half of all included requests, as it would not have been feasible to search for all 303 indications. Sixth, we did not consider a potential association of factors other than supporting RCT evidence on the reimbursement decision, such as treatment costs. Seventh, approval status may change over time; thus, some of the indications considered off-label in our study may have been approved later. However, there were no substantial changes in the off-label use reimbursement process in the Swiss health care system such that would limit the applicability of our findings.

## Conclusions

This cross-sectional study examined off-label reimbursement for cancer drugs in the Swiss health care system, which is characterized by high diversity, with more than 50 different health insurances in a federal system of 26 cantons, a strong private care sector, and high health care spending. In this system that enables the access to off-label use, off-label use was frequently used in the first-or second-line of cancer treatment and sometimes represented a new standard of care. The level of available evidence was not associated with reimbursement decision-making in our analysis, leaving clinicians and patients with high uncertainty and calls for immediate action to change the reimbursement process and guarantee fair access and evidence-based cancer care.

## Notes

Supplement. **eAppendix.** Supplementary Methods **eFigure 1.** Patient Flowchart and Selection Process **eFigure 2.** Most Frequently Requested Off-Label Use Indications **eTable 1.** Overview Off-Label Use Indications **eTable 2.** Overview of Evidence for Off-Label Use Indications **eTable 3.** Patient, Disease, and Treatment Characteristics of Reimbursement Requests and Their Associations With Reimbursement Decisions **eTable 4.** Sensitivity Analysis and Missing Imputation Calculations
