# Supplementary material for: Association of Supporting Trial Evidence and Reimbursement for Off-Label Use of Cancer Drugs
Source: JAMA Netw Open. 2021 Mar 2;4(3):e210380. doi: 10.1001/jamanetworkopen.2021.0380 (PMC7926292; doi:10.1001/jamanetworkopen.2021.0380)
Supplement: Supplement. — eAppendix. Supplementary Methods eFigure 1. Patient Flowchart and Selection Process eFigure 2. Most Frequently Requested Off-Label Use Indications eTable 1. Overview Off-Label Use Indications eTable 2. Overview of Evidence for Off-Label Use Indications eTable 3. Patient, Disease, and Treatment Characteristics of Reimbursement Requests and Their Associations With Reimbursement Decisions eTable 4. Sensitivity Analysis and Missing Imputation Calculations [file jamanetwopen-e210380-s001.pdf]

Supplemental Online Content

Herbrand AK, Schmitt AM, Briel M, et al. Association of supporting trial evidence and reimbursement for off-label use of cancer drugs. *JAMA Netw Open*. 2021;4(3):e210380. doi:10.1001/jamanetworkopen.2021.0380

eAppendix. Supplementary Methods

eFigure 1. Patient Flowchart and Selection Process

eFigure 2. Most Frequently Requested Off-Label Use Indications

eTable 1. Overview Off-Label Use Indications

eTable 2. Overview of Evidence for Off-Label Use Indications

eTable 3. Patient, Disease, and Treatment Characteristics of Reimbursement Requests and Their Associations With Reimbursement Decisions

eTable 4. Sensitivity Analysis and Missing Imputation Calculations

This supplemental material has been provided by the authors to give readers additional information about their work.

© 2021 Herbrand AK et al. *JAMA Network Open.*

eAppendix. Supplementary Methods

Detailed description of OLU conditions and definitions

We considered all requested drug treatments as OLU if there was at least one deviation from the Swissmedic drug label regarding the following four domains: disease (as defined by histology and biomarker), treatment setting (e.g. advanced or adjuvant), line of treatment (e.g. treatment naive patients), and use of the drug (e.g. as single agent or in combination, exposure to a specific drug in previous treatment). Specifically, we considered it OLU if at least one of the following conditions was fulfilled:

- Disease: drug use not approved for disease as defined by histology (e.g. squamous non-small cell adenocarcinoma of the lung); certain biomarker status not present (e.g. BRAF inhibitor for advanced non-BRAF mutant melanoma).
- Treatment setting: intended setting for either solid tumors (neoadjuvant, adjuvant, advanced) or hematological diseases (induction, maintenance, post-autoSCT, post-alloSCT, palliative); if a tumor was only borderline resectable/borderline advanced but the therapy was intended to be neoadjuvant, we counted it as neoadjuvant.
- Line of treatment: counted separately for adjuvant/neoadjuvant settings and advanced settings (e.g. first line advanced does not have to be first line in general; exception: if the study explicitly mentioned “untreated” patients or patients “that never received any chemotherapy”); we clearly differentiated between first and second line of treatment; second or third line of treatment and beyond could be summarized as relapsed if there was suitable evidence available that allowed us to do so; for hematological malignancies line of treatment was negligible in respect to the setting (e.g. for maintenance after autologous or allogeneic transplant the actual line of treatment was negligible).
- Drug use: single agent or combination therapy (if relevant also corticosteroids or hormonal therapy counted as combination therapy if used in a treatment scheme); always with the same drug as requested for conformity (e.g. FOLFIRI = Irinotecan+Combi); also we respected comments about previous therapy (e.g. “previously treated with platin” or “not previously treated with doxorubicin”).

© 2021 Herbrand AK et al. *JAMA Network Open.*

eFigure 1. Patient Flowchart and Selection Process

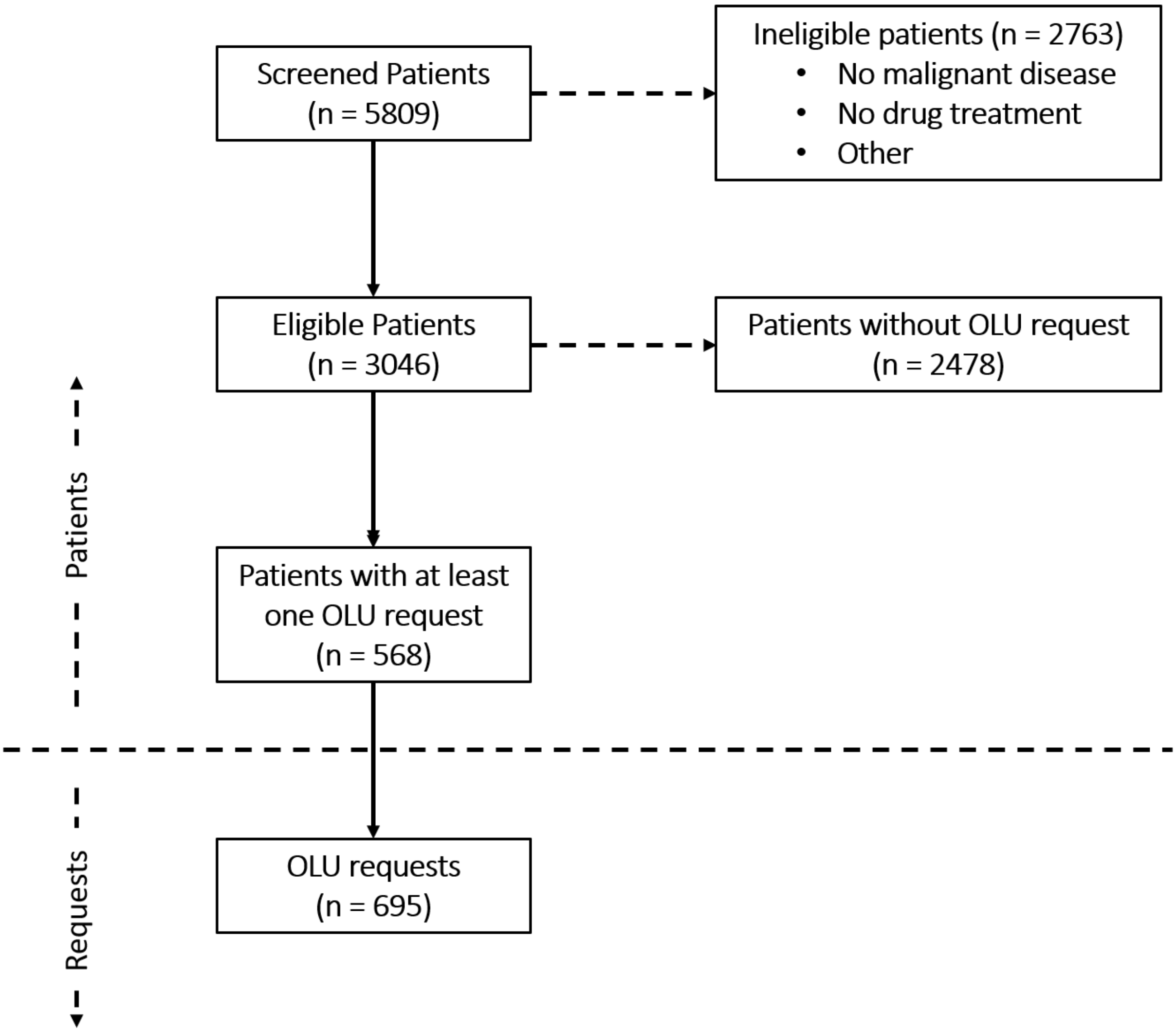

For 568 of 3046 eligible patients, at least one OLU request was issued with a total of 695 issued requests.

© 2021 Herbrand AK et al. *JAMA Network Open.*

eFigure 2. Most Frequently Requested Off-Label Use Indications

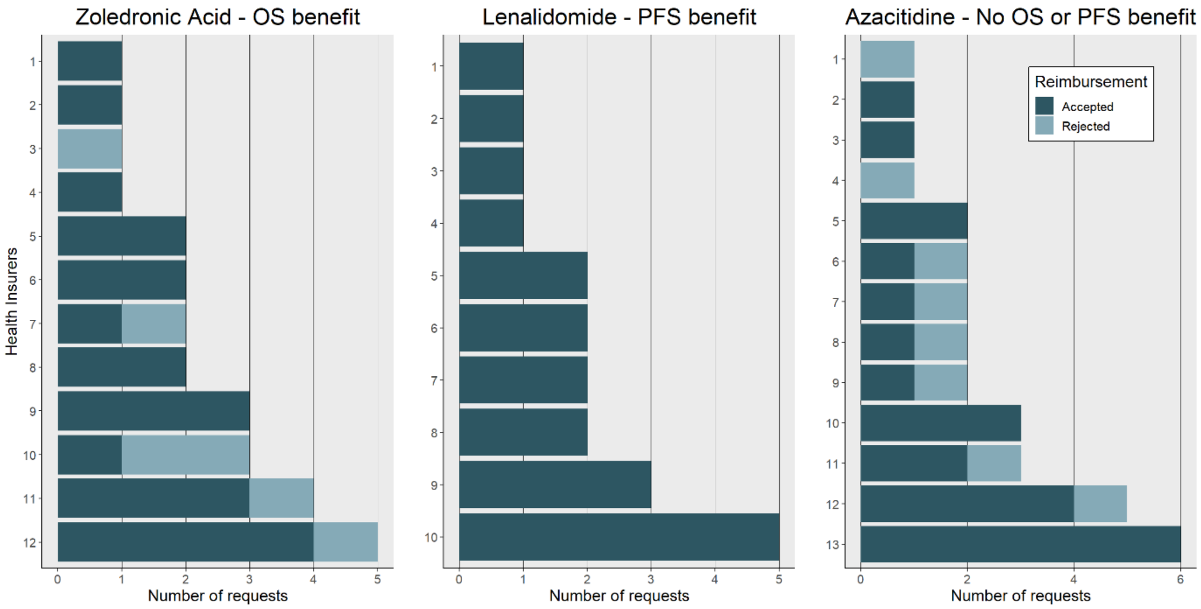

<u>**eFigure 2 a)-c):**</u> Reimbursement requests stratified by acceptance or rejection for the three most frequently requested indications (Zoledronic acid given during adjuvant treatment for hormone-positive breast cancer, lenalidomide as maintenance therapy after autologous stem cell transplantation in multiple myeloma, and azacitidine as maintenance after allogeneic stem cell transplantation in acute myeloid leukemia or myelodysplastic syndrome) for each health insurer (coded by a number on the left).

Patients with the same disease and treatment indication within the same health insurer sometimes receive access and sometimes not, based on inconsistent reimbursement decisions. The Figure illustrates that OLU indications for which only evidence for progression-free survival benefit exists may be reimbursed 100%, whereas OLU indications with proven benefit for overall survival may sometimes be rejected.

Abbreviations: OS – Overall survival; PFS-Progression-free survival;

© 2021 Herbrand AK et al. *JAMA Network Open.*

eTable 1. Overview Off-Label Use Indications

Drug	Disease	Treatment Setting	Line of Treatment	Application	Requests
Azacitidine	AML & MDS	Post-allo	1st	Single agent	31
Zoledronic acid	Breast Cancer	Adjuvant	2nd	Drug combination	28
Lenalidomide	Multiple Myeloma	Post-auto	2nd	Single agent	26
Nivolumab	Melanoma	Adjuvant	1st	Single agent	16
Abiraterone	Prostate Cancer	Advanced	1st	Drug combination	14
Lomustin	Glioblastoma	Advanced	2nd, 3rd or beyond	Single agent	13
Rituximab	Follicular Lymphoma	Palliative	1st	Single agent	11
Bortezomib	Multiple Myeloma	Induction	1st	Drug combination	9
Capecitabine	Pancreatic cancer	Adjuvant	1st	Drug combination	9
Ipilimumab	SCLC	Advanced	2nd, 3rd or beyond	Drug combination	9
Nab paclitaxel	Pancreatic cancer	Advanced	2nd, 3rd or beyond	Drug combination	9
Pembrolizumab	NSCLC, Adenocarcinoma	Advanced	1st	Drug combination	9
Rituximab	Posttransplant lymphoproliferative disorder	Induction	1st	Single agent	9
Sorafenib	AML	Post-allo SCT	2nd, 3rd line or beyond	Single agent	9
Atezolizumab	Urothelial Cancer	Advanced	1st	Single agent	8
Olaratumab	Soft Tissue Sarcoma	Advanced	3rd or beyond	Drug combination	8
Pertuzumab	Breast Cancer	Neoadjuvant	1st	Drug combination	8
Sorafenib	AML	Post-allo SCT	2nd	Drug combination	8
Bevacizumab	Colorectal Cancer	Advanced	3rd or beyond	Drug combination	7
Novo-TTF	Glioblastoma	Advanced	1st	Drug combination	7
Pembrolizumab	NSCLC, NOS	Advanced	1st	Single agent	7
Irinotecan	Gastro-Esophageal Junction, adenocarcinoma	Advanced	2nd	Drug combination	6
Nivolumab	Squamous Cell Carcinoma Of The Skin	Advanced	3rd or beyond	Single agent	6
Osimertinib	NSCLC, Adenocarcinoma	Advanced	2nd	Single agent	6
Ipilimumab	Melanoma	Adjuvant	1st	Single agent	5
Irinotecan	Pancreatic cancer	Advanced	2nd	Drug combination	5
Obinutuzumab	DLBCL	Palliative	2nd	Drug combination	5
Osimertinib	NSCLC, Adenocarcinoma	Advanced	2nd	Single agent	5
Pembrolizumab	Urothelial Cancer	Advanced	2nd	Single agent	5

© 2021 Herbrand AK et al. *JAMA Network Open.*

Pembrolizumab	SCLC	Advanced	3rd or beyond	Single agent	5
Ramucirumab	NSCLC, Adenocarcinoma	Advanced	3rd or beyond	Drug combination	5
Rituximab	Follicular Lymphoma	Maintenance	1st	Single agent	5
Temozolomide	Brain Tumor WHO Grading II	Advanced	1st	Single agent	5
Bendamustine	Marginal zone lymphoma	Induction	1st	Drug combination	4
Bevacizumab	Mesothelioma	Advanced	1st	Drug combination	4
Bevacizumab	NSCLC, Adenocarcinoma	Advanced	2nd	Drug combination	4
Bevacizumab	Brain Tumor WHO Grading III	Advanced	2nd	Single agent	4
Crizotinib	NSCLC, Adenocarcinoma	Advanced	1st	Single agent	4
Lomustin	Brain Tumor WHO Grading III	Advanced	2nd	Single agent	4
Nivolumab	Hodgkin Lymphoma	Palliative	3rd or beyond	Single agent	4
Pembrolizumab	Urothelial Cancer	Advanced	1st	Single agent	4
Regorafenib	HCC	Advanced	2nd	Single agent	4
Thiotepa	Primary central nervous system Lymphoma	Induction	1st	Drug combination	4
Atezolizumab	Urothelial Cancer	Advanced	2nd	Single agent	3
Bexaroten	Cutaneous T-Cell lymphoma	Induction	2nd	Single agent	3
Capecitabine	Cholangiocellular Carcinoma	Adjuvant	1st	Single agent	3
Cetuximab	Head and Neck	Advanced	1st	Drug combination	3
Crizotinib	NSCLC, Adenocarcinoma	Advanced	3rd or beyond	Single agent	3
Daratumumab	Multiple Myeloma	Palliative	3rd or beyond	Single agent	3
Denosumab	Multiple Myeloma	Palliative	2nd	Single agent	3
Docetaxel	Prostate Cancer	Advanced	1st	Drug combination	3
Doxorubicin liposomal	DLBCL	Induction	1st	Drug combination	3
Exemestan	Breast Cancer	Adjuvant	1st	Drug combination	3
Irinotecan	Pancreatic cancer	Advanced	2nd	Drug combination	3
Irinotecan	Pancreatic cancer	Neoadjuvant	1st	Drug combination	3
Lomustin	Glioblastoma	Advanced	2nd	Drug combination	3
Midostaurin	AML	Post-allo	3rd or beyond	Single agent	3
Nivolumab	HCC	Advanced	2nd	Single agent	3
Nivolumab	SCLC	Advanced	3rd or beyond	Single agent	3
Novo-TTF	Glioblastoma	Advanced	2nd	Single agent	3
Obinutuzumab	CLL/SLL	Palliative	3rd or beyond	Drug combination	3

© 2021 Herbrand AK et al. *JAMA Network Open.*

Pembrolizumab	Colorectal Cancer	Advanced	3rd or beyond	Single agent	3
Temozolomide	Neuro-Endocrine Tumour	Advanced	1st	Drug combination	3
Temozolomide	Neuro-Endocrine Tumour	Advanced	2nd	Drug combination	3
Arsenic trioxide	Acute Promyelocyte Leukemia	Induction	1st	Drug combination	2
Bendamustine	M. Waldenström	Induction	1st	Drug combination	2
Bevacizumab	NSCLC, Adenocarcinoma	Advanced	1st	Drug combination	2
Bortezomib	Multiple Myeloma	Induction	1st	Drug combination	2
Capecitabine	Pancreatic cancer	Advanced	3rd or beyond	Drug combination	2
Carfilzomib	Multiple Myeloma	Induction	1st	Drug combination	2
Cetuximab	NSCLC, Adenocarcinoma	Advanced	3rd or beyond	Drug combination	2
Cetuximab	Squamous Cell Carcinoma Of The Skin	Advanced	1st	Single agent	2
Dabrafenib	Melanoma	Adjuvant	1st	Drug combination	2
Dasatinib	CML	Maintenance	2nd	Single agent	2
Decitabine	MDS	Palliative	2nd	Single agent	2
Doxorubicin liposomal	Soft Tissue Sarcoma	Advanced	1st	Single agent	2
Eribulin mesylate	Soft Tissue Sarcoma	Advanced	3rd or beyond	Single agent	2
Gemcitabine	Soft Tissue Sarcoma	Advanced	3rd or beyond	Drug combination	2
Imatinib mesylate	ALL	Post-allo	2nd, 3rd or beyond	Single agent	2
Lenalidomide	DLBCL	Palliative	3rd or beyond	Single agent	2
Nivolumab	NSCLC, Squamous	Advanced	1st	Single agent	2
Olaparib	Breast Cancer	Advanced	2nd, 3rd or beyond	Single agent	2
Olaparib	Osteosarcoma	Advanced	2nd, 3d or beyond	Single agent	2
Paclitaxel	Gastro-Esophageal Junction, adenocarcinoma	Neoadjuvant	1st	Drug combination	2
Peginterferon alpha 2a	Primary Myelofibrosis	Induction	2nd	Single agent	2
Pembrolizumab	Gastric Cancer	Advanced	2nd, 3rd or beyond	Single agent	2
Pembrolizumab	Melanoma	Advanced	1st	Single agent	2
Pembrolizumab	NSCLC, Squamous	Advanced	1st	Single agent	2
Rituximab	Marginal zone lymphoma	Induction	1st	Drug combination	2
Rituximab	ALL	Induction	1st	Drug combination	2
Rituximab	DLBCL	Induction	1st	Single agent	2
Rituximab	Hodgkin Lymphoma	Induction	1st	Drug combination	2

Rituximab	Mantle Cell Lymphoma	Induction	1st	Drug combination	2
Rituximab	Mantle Cell Lymphoma	Post-auto	2nd	Single agent	2
Rituximab	Marginal zone lymphoma	Advanced	1st	Single agent	2
Temozolomide	Medulloblastoma	Advanced	2nd, 3rd or beyond	Single agent	2
Trabectedin	Soft Tissue Sarcoma	Advanced	2nd	Single agent	2
Trifluridine; tipiracil	Colorectal Cancer	Advanced	2nd, 3rd or beyond	Single agent	2
Vinflunin	Urothelial Cancer	Advanced	2nd	Single agent	2
Abiraterone	Head and Neck	Advanced	2nd	Drug combination	1
Afatinib	Head and Neck	Advanced	3rd or beyond	Single agent	1
Alemtuzumab	T-Cell Leukemia	Induction	1st	Single agent	1
Alitretinoin	AML	Palliative	3rd or beyond	Single agent	1
Atezolizumab	Anal cancer	Advanced	2nd	Single agent	1
Atezolizumab	Colorectal Cancer	Advanced	3rd or beyond	Drug combination	1
Atezolizumab	Germ Cell Tumor of Men	Advanced	1st	Single agent	1
Azacitidine	CML	Post-allo	3rd or beyond	Single agent	1
Azacitidine	CMML	Induction	1st	Single agent	1
Azacitidine	CMML	Post-allo	3rd or beyond	Single agent	1
Azacitidine	MDS	Palliative	2nd	Single agent	1
Bendamustine	CLL/SLL	Induction	1st	Drug combination	1
Bendamustine	DLBCL	Induction	1st	Drug combination	1
Bendamustine	DLBCL	Induction	3rd or beyond	Drug combination	1
Bendamustine	Hodgkin Lymphoma	Palliative	3rd or beyond	Single agent	1
Bendamustine	Mantle Cell Lymphoma	Induction	2nd	Drug combination	1
Bendamustine	Marginal zone lymphoma	Induction	3rd or beyond	Drug combination	1
Bendamustine	Multiple Myeloma	Palliative	3rd or beyond	Drug combination	1
Bevacizumab	Glioblastoma	Advanced	1st	Single agent	1
Bevacizumab	Glioblastoma	Advanced	2nd	Drug combination	1
Bevacizumab	NSCLC, Adenocarcinoma	Advanced	3rd or beyond	Drug combination	1
Bevacizumab	Renal Cell Carcinoma	Neoadjuvant	1st	Single agent	1
Bexaroten	Peripheral T-Cell Lymphoma	Palliative	2nd	Single agent	1
Bortezomib	Multiple Myeloma	Induction, Maintenance	3rd or beyond	Drug combination	1
Bortezomib	Multiple Myeloma	Post-auto	3rd or beyond	Drug combination	1
Brentuximab vedotin	Cutaneous T-Cell lymphoma	Palliative	3rd or beyond	Single agent	1
Brentuximab vedotin	Hodgkin Lymphoma	Induction	1st	Unclear	1

Brentuximab vedotin	Peripheral T-Cell Lymphoma	Palliative	3rd or beyond	Single agent	1
Brentuximab vedotin	T-Cell Lymphoma	Palliative	2nd	Single agent	1
Brigatinib	NSCLC, Adenocarcinoma	Advanced	3rd or beyond	Drug combination	1
Cabozantinib	Renal Cell Carcinoma	Advanced	3rd or beyond	Single agent	1
Cabozantinib	Undifferentiated Neoplasm	Advanced	3rd or beyond	Single agent	1
Capecitabine	Cholangiocellular Carcinoma	Advanced	2nd	Single agent	1
Capecitabine	Colorectal Cancer	Neoadjuvant	1st	Single agent	1
Capecitabine	Gastric Cancer	Perioperative	1st	Drug combination	1
Capecitabine	Gastro-Esophageal Junction, adenocarcinoma	Neoadjuvant	1st	Drug combination	1
Capecitabine	Urothelial Cancer	Adjuvant	1st	Drug combination	1
Carfilzomib	Multiple Myeloma	Post-auto, Palliative	3rd or beyond	Drug combination	1
Ceritinib	NSCLC, Adenocarcinoma	Advanced	3rd or beyond	Single agent	1
Cetuximab	Head and Neck	Advanced	2nd	Drug combination	1
Cetuximab	Head and Neck	Advanced	3rd or beyond	Single agent	1
Cobimetinib	Melanoma	Advanced	1st	Single agent	1
Crizotinib	Cancer of unkown primary	Advanced	2nd	Single agent	1
Crizotinib	Gastric Cancer	Advanced	3rd or beyond	Single agent	1
Crizotinib	Gastro-Esophageal Junction, adenocarcinoma	Advanced	3rd or beyond	Single agent	1
Cytarabine liposomal	Breast Cancer	Advanced	1st	Single agent	1
Cytarabine liposomal	DLBCL	Induction	2nd	Single agent	1
Dabrafenib	NSCLC, Adenocarcinoma	Advanced	2nd	Drug combination	1
Daratumumab	Multiple Myeloma	Induction	1st	Single agent	1
Daratumumab	Multiple Myeloma	Post-auto	3rd or beyond	Drug combination	1
Daratumumab	T-Cell Lymphoma	Palliative	2nd	Single agent	1
Dasatinib	ALL	Induction	2nd	Single agent	1
Degarelix acetate	Breast Cancer	Advanced	2nd	Drug combination	1
Denosumab	Breast Cancer	Adjuvant	2nd	Drug combination	1
Docetaxel	Esophageal Cancer, squamous	Advanced	2nd	Single agent	1
Docetaxel	Gastro-Esophageal Junction, adenocarcinoma	Advanced	3rd or beyond	Single agent	1
Doxorubicin liposomal	Breast Cancer	Adjuvant	1st	Drug combination	1
Doxorubicin liposomal	Cutaneous T-Cell lymphoma	Palliative	2nd	Single agent	1
Doxorubicin liposomal	Glioblastoma	Advanced	3rd or beyond	Single agent	1

© 2021 Herbrand AK et al. *JAMA Network Open.*

Doxorubicin liposomal	Multiple Myeloma	Palliative	3rd or beyond	Drug combination	1
Doxorubicin liposomal	Soft Tissue Sarcoma	Advanced	2nd	Single agent	1
Enzalutamide	Prostate Cancer	Advanced	3rd or beyond	Single agent	1
Erlotinib	Renal Cell Carcinoma	Advanced	2nd	Drug combination	1
Everolimus	Undifferentiated Neoplasm	Advanced	1st	Drug combination	1
Exemestan	Breast Cancer	Adjuvant	3rd or beyond	Single agent	1
Gemcitabine	Adrenocortical carcinoma	Advanced	2nd	Drug combination	1
Gemcitabine	Cholangiocellular Carcinoma	Advanced	1st	Drug combination	1
Gemcitabine	Cholangiocellular Carcinoma	Advanced	2nd	Drug combination	1
Gemcitabine	HCC	Advanced	1st	Drug combination	1
Gemcitabine	HCC	Advanced	2nd	Drug combination	1
Gemcitabine	Head and Neck	Advanced	3rd or beyond	Single agent	1
Gemtuzumab ozogamicin	AML	Post-allo	2nd	Single agent	1
Goserelin acetate	Head and Neck	Advanced	1st	Drug combination	1
Ibritumomab tiuxetan	Mantle Cell Lymphoma	Maintenance	3rd or beyond	Single agent	1
Ibrutinib	Marginal zone lymphoma	Induction	3rd or beyond	Single agent	1
Ibrutinib	Pancreatic cancer	Advanced	3rd or beyond	Drug combination	1
Idelalisib	Marginal zone lymphoma	Palliative	3rd or beyond	Drug combination	1
Imatinib mesylate	ALL	Induction	1st	Drug combination	1
Imatinib mesylate	Chordom	Advanced	1st	Single agent	1
Inotuzumab ozogamicin	ALL	Induction	1st	Drug combination	1
Inotuzumab ozogamicin	Burkitt Lymphoma	Palliative	3rd or beyond	Single agent	1
Ipilimumab	Head and Neck	Advanced	3rd or beyond	Drug combination	1
Ipilimumab	NSCLC, Adenocarcinoma	Advanced	3rd or beyond	Drug combination	1
Ipilimumab	Renal Cell Carcinoma	Advanced	1st	Drug combination	1
Irinotecan	Gastric Cancer	Advanced	1st	Drug combination	1
Irinotecan	Gastro-Esophageal Junction, adenocarcinoma	Advanced	3rd or beyond	Single agent	1
Irinotecan	Urothelial Cancer	Advanced	3rd or beyond	Drug combination	1
Ixazomib	Multiple Myeloma	Post-auto	3rd or beyond	Drug combination	1

Ixazomib	Multiple Myeloma	Palliative	3rd or beyond	Drug combination	1
Lenalidomide	Anaplastic large-cell lymphoma	Other, Palliative	3rd or beyond	Single agent	1
Lenalidomide	Cutaneous T-Cell lymphoma	Palliative	3rd or beyond	Single agent	1
Lenalidomide	DLBCL	Maintenance	3rd or beyond	Single agent	1
Lenalidomide	DLBCL	Palliative	3rd or beyond	Drug combination	1
Lenalidomide	Hodgkin Lymphoma	Maintenance	3rd or beyond	Single agent	1
Lenalidomide	Marginal zone lymphoma	Palliative	2nd	Single agent	1
Lenalidomide	MDS	Induction	2nd	Single agent	1
Lenalidomide	Multiple Myeloma	Induction	2nd	Single agent	1
Lenalidomide	Multiple Myeloma	Maintenance	1st	Single agent	1
Lenalidomide	Multiple Myeloma	Maintenance	2nd	Drug combination	1
Lenalidomide	Multiple Myeloma	Post-allo	3rd or beyond	Single agent	1
Lenalidomide	Multiple Myeloma	Post-auto, Other	1st	Drug combination	1
Lenalidomide	Multiple Myeloma	Post-auto	3rd or beyond	Drug combination	1
Lomustin	Brain Tumor WHO Grading III	Advanced	1st	Drug combination	1
Lu-177-PSMA	Prostate Cancer	Advanced	3rd or beyond	Single agent	1
Midostaurin	AML	Palliative	3rd or beyond	Single agent	1
Mifamurtid	Osteosarcoma	Advanced	2nd	Drug combination	1
Mitotane	Adrenocortical carcinoma	Adjuvant	1st	Single agent	1
Nelfinavir	Multiple Myeloma	Induction	2nd	Drug combination	1
Nelfinavir	Multiple Myeloma	Palliative	3rd or beyond	Drug combination	1
Nilotinib	ALL	Induction	1st	Drug combination	1
Nilotinib	ALL	Other	2nd	Single agent	1
Nintedanib	NSCLC, Adenocarcinoma	Advanced	3rd or beyond	Drug combination	1
Nivolumab	Gastro-Esophageal Junction, adenocarcinoma	Advanced	3rd or beyond	Single agent	1
Nivolumab	Glioblastoma	Advanced	3rd or beyond	Single agent	1
Nivolumab	Head and Neck	Advanced	1st	Single agent	1
Nivolumab	Melanoma	Advanced	1st	Single agent	1
Nivolumab	Melanoma	Advanced	2nd	Single agent	1
Nivolumab	NSCLC, Large Cell	Advanced	2nd	Single agent	1
Nivolumab	NSCLC, NOS	Advanced	1st	Single agent	1
Nivolumab	Squamous Cell Carcinoma Of The Skin	Advanced	2nd	Single agent	1
Nivolumab	Undifferentiated Neoplasm	Advanced	2nd	Single agent	1

Olaratumab	Soft Tissue Sarcoma	Advanced	2nd	Single agent	1
Oxaliplatin	Gastric Cancer	Adjuvant	1st	Drug combination	1
Oxaliplatin	Gastric Cancer	Perioperative	1st	Drug combination	1
Paclitaxel	Cancer of unkown primary	Advanced	1st	Drug combination	1
Paclitaxel	Esophageal Cancer, squamous	Advanced	3rd or beyond	Single agent	1
Paclitaxel	Soft Tissue Sarcoma	Advanced	1st	Single agent	1
Paclitaxel	Urothelial Cancer	Advanced	2nd	Drug combination	1
Palbociclib	Germ Cell Tumor of Men	Advanced	2nd	Single agent	1
Palbociclib	Soft Tissue Sarcoma	Advanced	1st	Single agent	1
Panitumumab	Colorectal Cancer	Advanced	1st	Single agent	1
Panitumumab	Colorectal Cancer	Advanced	3rd or beyond	Drug combination	1
Panobinostat	Multiple Myeloma	Palliative	3rd or beyond	Single agent	1
Peginterferon alpha 2a	Essential thrombocythemia	Induction	1st	Single agent	1
Peginterferon alpha 2a	Essential thrombocythemia	Induction	2nd	Single agent	1
Peginterferon alpha 2a	Polycythemia vera	Palliative	1st	Single agent	1
Peginterferon alpha 2a	Primary Myelofibrosis	Induction	1st	Single agent	1
Pembrolizumab	NSCLC, Squamous	Advanced	1st	Drug combination	1
Pembrolizumab	Anal cancer	Advanced	1st	Single agent	1
Pembrolizumab	Anal cancer	Advanced	2nd	Single agent	1
Pembrolizumab	Breast Cancer	Advanced	2nd	Single agent	1
Pembrolizumab	Cutaneous T-Cell lymphoma	Palliative	3rd or beyond	Single agent	1
Pembrolizumab	Endometrial cancer	Advanced	3rd or beyond	Single agent	1
Pembrolizumab	Esophageal Cancer, denocarcinoma	Advanced	2nd	Single agent	1
Pembrolizumab	Head and Neck	Advanced	2nd	Single agent	1
Pembrolizumab	Head and Neck	Other	1st	Single agent	1
Pembrolizumab	Melanoma	Adjuvant	1st	Single agent	1
Pembrolizumab	Melanoma	Advanced	2nd	Drug combination	1
Pembrolizumab	Melanoma	Advanced	2nd	Single agent	1
Pembrolizumab	Merkel cell carcinoma	Advanced	1st	Single agent	1
Pembrolizumab	Mesothelioma	Advanced	3rd or beyond	Single agent	1
Pembrolizumab	Multiple Myeloma	Palliative	3rd or beyond	Single agent	1
Pembrolizumab	NSCLC, Adenocarcinoma	Advanced	2nd	Single agent	1
Pembrolizumab	NSCLC, Adenocarcinoma	Advanced	3rd or beyond	Drug combination	1
Pembrolizumab	Pancreatic cancer	Advanced	2nd	Single agent	1
Pembrolizumab	Soft Tissue Sarcoma	Advanced	2nd	Single agent	1
Pembrolizumab	Squamous Cell Carcinoma Of The Skin	Advanced	2nd	Single agent	1

© 2021 Herbrand AK et al. *JAMA Network Open.*

Pemetrexed	Mesothelioma	Neoadjuvant	1st	Drug combination	1
Pemetrexed	NSCLC, Adenocarcinoma	Advanced	1st	Single agent	1
Pemetrexed	NSCLC, Adenocarcinoma	Advanced	2nd	Single agent	1
Pemetrexed	NSCLC, Adenocarcinoma	Advanced	3rd or beyond	Drug combination	1
Pemetrexed	Urothelial Cancer	Advanced	2nd	Single agent	1
Pertuzumab	Breast Cancer	Adjuvant	1st	Drug combination	1
Pertuzumab	Breast Cancer	Adjuvant	2nd	Drug combination	1
Pertuzumab	Breast Cancer	Advanced	1st	Drug combination	1
Pertuzumab	Breast Cancer	Advanced	3rd or beyond	Drug combination	1
Pidilizumab	DLBCL	Post-auto	3rd or beyond	Single agent	1
Ponatinib	ALL	Other	Unclear	Single agent	1
Pralatrexate	Cutaneous T-Cell lymphoma	Palliative	3rd or beyond	Single agent	1
Ramucirumab	Gastric Cancer	Advanced	2nd	Drug combination	1
Rituximab	ALL	Maintenance	2nd	Single agent	1
Rituximab	Burkitt Lymphoma	Induction	1st	Drug combination	1
Rituximab	Burkitt Lymphoma	Induction	2nd	Drug combination	1
Rituximab	CLL/SLL	Induction	1st	Drug combination	1
Rituximab	DLBCL	Induction	2nd	Single agent	1
Rituximab	M. Waldenström	Post-auto	3rd or beyond	Single agent	1
Rituximab	Mantle Cell Lymphoma	Maintenance	2nd	Single agent	1
Rituximab	Marginal zone lymphoma	Induction	1st	Single agent	1
Romidepsin	Cutaneous T-Cell lymphoma	Palliative	3rd or beyond	Single agent	1
Romidepsin	Peripheral T-Cell Lymphoma	Induction	2nd	Single agent	1
Sipuleucel-t	Prostate Cancer	Advanced	3rd or beyond	Single agent	1
Sorafenib	AML	Maintenance	2nd	Single agent	1
Sorafenib	AML	Other	1st	Single agent	1
Streptozocin	Adrenocortical carcinoma	Advanced	3rd or beyond	Single agent	1
Temozolomide	Ependymoma	Advanced	1st	Single agent	1
Temozolomide	Glioblastoma	Advanced	1st	Single agent	1
Temozolomide	Melanoma	Advanced	3rd or beyond	Single agent	1
Temozolomide	NSCLC, Adenocarcinoma	Advanced	3rd or beyond	Drug combination	1
Temozolomide	Soft Tissue Sarcoma	Advanced	Unclear	Drug combination	1
Trabectedin	Soft Tissue Sarcoma	Adjuvant	1st	Single agent	1
Trabectedin	Soft Tissue Sarcoma	Advanced	1st	Single agent	1

© 2021 Herbrand AK et al. *JAMA Network Open.*

Trabectedin	Soft Tissue Sarcoma	Advanced	3rd or beyond	Single agent	1
Trastuzumab	Breast Cancer	Adjuvant	1st	Single agent	1
Trastuzumab	Breast Cancer	Advanced	2nd	Drug combination	1
Trastuzumab	Cholangiocellular Carcinoma	Advanced	2nd	Single agent	1
Trastuzumab	Gastric Cancer	Advanced	2nd	Drug combination	1
Trastuzumab emtansine	Breast Cancer	Advanced	2nd	Single agent	1
TRASTUZUMAB s.c.	Breast Cancer	Advanced	1st	Drug combination	1
Triptorelin pamoate	Breast Cancer	Adjuvant	1st	Drug combination	1
Vemurafenib	NSCLC, Adenocarcinoma	Advanced	2nd	Single agent	1
Venetoclax	CLL/SLL	Palliative	3rd or beyond	Single agent	1
Venetoclax	T-Cell Leukemia	Induction	3rd or beyond	Single agent	1
Vincristin	Brain Tumor WHO Grading II	Advanced	1st	Drug combination	1
Zoledronic acid	Breast Cancer (post-menopausal)	Adjuvant	1st	Drug combination	1

Abbreviations: AML = Acute myeloid leukemia; MDS = Myelodysplastic syndrome; Post-allo = Post allogeneic stem cell transplantation; Post-auto = Post-autologous stem cell transplantation; NSCLC = Non-small cell lung cancer; SCLC = Small-cell lung cancer; DLBCL = Diffuse large B-cell lymphoma; HCC = Hepatocellular carcinoma; CLL = Chronic lymphocytic leukemia; SLL = Small lymphocytic lymphoma; CML = Chronic myelogenous leukemia; ALL = Acute lymphoblastic leukemia; CMML = Chronic myelomonocytic leukemia; s.c. = subcutaneous;

© 2021 Herbrand AK et al. *JAMA Network Open.*

eTable 2. Overview of Evidence for Off-Label Use Indications

Drug	OLU Indication	Author (Study)	Publication Date	Publication Type	HR for OS (95% CIs)	Cumulative HR for OS (95% CIs)	HR for PFS (95% CIs)	Cumulative HR for PFS (95% CIs)	Category of Clinical benefit
Abiraterone	Prostate Cancer, Advanced, 1st line, Drug combination	James (STAMPEDE (Arms A versus G))	2017-06-03	First full publication	0.61 (0.49-0.75)	-	0.31 (0.26-0.37)	-	OS
		Fizazi (LATITUDE)	2017-06-04	First full publication	0.62 (0.51-0.76)	0.62 (0.53-0.71)	0.47 (0.4-0.56)	0.38 (0.25-0.57)	OS
Atezolizumab	Urothelial Cancer, Advanced, 1st line, Single agent	-	-	-	-	-	-	-	No RCT
Atezolizumab	Urothelial Cancer, Advanced, 2nd line, Single agent	Powles (IMvigor211)	2017-12-18	First full publication	0.85 (0.73-0.99)	-	-	-	OS
Azacitidine	AML & MDS, Post-allo, NA, Single agent	-	-	-	-	-	-	-	No RCT
Bendamustine	Marginal zone lymphoma, Induction, 1st line, Drug combination	Rummel (NHL 1-2003)	2013-02-20	First full publication	-	-	0.7 (0.34-1.43)	-	No Benefit
Bevacizumab	Mesothelioma, Advanced, 1st line, Drug combination	Zalcman (MAPS)	2015-12-21	First full publication	0.77 (0.62-0.95)	-	0.61 (0.5-0.75)	-	OS
Bevacizumab	NSCLC, Adenocarcinoma, Advanced, 2nd line, 3rd line or	-	-	-	-	-	-	-	No RCT

© 2021 Herbrand AK et al. *JAMA Network Open.*

	beyond, Drug combination								
Bevacizumab	Brain Tumor WHO Grading III, Advanced, 2nd line, Single agent	-	-	-	-	-	-	-	No RCT
Bevacizumab	Colorectal Cancer, Advanced, 3rd line or beyond, Drug combination	-	-	-	-	-	-	-	No RCT
Bexaroten	Cutaneous T-Cell lymphoma, Induction, 2nd line, Single agent	Whittaker (EORTC 21011)	2012-09-01	First full publication	-	-	-	-	No RCT
Bortezomib	Multiple Myeloma, Induction, 1st line, Drug combination (VTD)	Rosinol (GEM05MENOS65)	2012-07-12	First full publication	-	-	-	-	No Benefit
		Cavo (Gimema-MMY-3006 Study)	2013-01-01	Abstract	-	-	0.66 (0.52-0.84)	-	PFS
		Moreau, (IFM2013-04)	2015-12-06	Abstract	-	-	-	0.66 (0.52-0.84)	PFS
		Moreau (IFM2013-04)	2016-03-21	First full publication	-	-	-	0.66 (0.52-0.84)	PFS
Bortezomib	Multiple Myeloma, Induction, 1st line, Drug combination (VRD)	Durie, (SWOG S0777)	2016-12-05	Abstract	0.67 (0.49-0.91)	-		-	OS

© 2021 Herbrand AK et al. *JAMA Network Open.*

		Durie (SWOG S0777)	2016-12-23	First full publication	0.71 (0.52-0.96)	-	-	-	OS
Capecitabine	Pancreatic cancer, Adjuvant, 1st line, Drug combination	Neoptolemos (ESPAC-4)	2016-06-20	Abstract	0.82 (0.68-0.98)	-	-	-	OS
		Neoptolemos (ESPAC-4)	2017-03-11	First full publication	0.82 (0.68-0.98)	-	-	-	OS
Capecitabine	Cholangiocellular Carcinoma, Adjuvant, 1st line, Single agent	Primrose (BILCAP)	2017-05-30	Abstract	0.8 (0.63-1.04)	-	-	-	No Benefit
Cetuximab	Head and Neck, Advanced, 1st line, Drug combination	-	-	-	-	-	-	-	No RCT
Crizotinib	NSCLC, Adenocarcinoma, Advanced, 1st line, Single agent	Solomon (PROFILE 1014)	2014-12-04	First full publication	0.82 (0.54-1.26)	-	0.45 (0.3-0.95)	-	PFS
Crizotinib	NSCLC, Adenocarcinoma, Advanced, 2nd line, 3rd line or beyond, Single agent	-	-	-	-	-	-	-	No RCT
Daratumumab	Multiple Myeloma, Palliative, 3rd line or beyond, Single agent	-	-	-	-	-	-	-	No RCT
Denosumab	Multiple Myeloma, Palliative, 2nd line, 3rd line or	Raje (NCT01345019)	2018-02-09	First full publication	-	-	-	-	No RCT

© 2021 Herbrand AK et al. *JAMA Network Open.*

	beyond, Single agent								
Docetaxel	Prostate Cancer, Advanced, 1st line, Drug combination	Gravis (GETUG-AFU15)	2013-01-08	First full publication	1.01 (0.75-1.36)	-	0.72 (0.57-0.91)	-	PFS
		Sweeney (CHAARTED)	2014-06-20	Abstract	0.63 (0.48-0.82)	0.79 (0.50-1.26)	-	0.72 (0.57-0.91)	PFS
		James (STAMPEDE)	2015-05-20	Abstract	0.76 (0.63-0.91)	0.78 (0.62-0.98)	-	0.72 (0.57-0.91)	OS
		Sweeney (CHAARTED)	2015-08-05	First full publication	0.61 (0.47-0.8)	0.77 (0.60-0.99)	0.61 (0.51-0.72)	0.65 (0.55-0.76)	OS
		Gravis (GETUG-AFU15)	2015-11-21	Updated analysis	0.88 (0.68-1.14)	0.75 (0.62-0.90)	0.67 (0.54-0.84)	0.63 (0.55-0.72)	OS
		James (STAMPEDE)	2015-12-21	First full publication	0.78 (0.66-0.93)	0.75 (0.63-0.91)	0.61 (0.53-0.79)	0.62 (0.56-0.70)	OS
Doxorubicin liposomal	DLBCL, Induction, 1st line, Drug combination	-	-	-	-	-	-	-	No RCT
Exemestan	Breast Cancer, Adjuvant, 1st line, Drug combination	Pagani (TEXT/SOFT)	2014-06-01	First full publication	1.14 (0.86-1.51)	-	0.72 (0.6-0.85)	-	PFS
Ipilimumab	SCLC, Advanced, 2nd line, 3rd line or beyond, Drug combination	Antonia (CheckMate 032)	2016-06-04	First full publication	-	-	-	-	No RCT
Ipilimumab	Melanoma, Adjuvant, 1st line, Single agent	Eggermont (EORTC 18071)	2014-09-27	Abstract	-	-	0.75 (0.64-0.9)	-	PFS

© 2021 Herbrand AK et al. *JAMA Network Open.*

		Eggermont (EORTC 18071)	2015-03-31	First full publication	-	-	0.75 (0.64-0.9)	-	PFS
		Eggermont (EORTC 18071)	2016-10-07	Updated analysis	0.72 (0.58-0.88)	-	0.76 (0.64-0.89)	-	PFS
Irinotecan	Pancreatic cancer, Advanced, 1st line, Drug combination	Conroy (NCT00112658)	2011-05-12	First full publication	0.57 (0.45-0.73)	-	0.47 (0.37-0.59)	-	OS
Irinotecan	Esophageal Cancer, adenocarcinoma, Advanced, 2nd line, 3rd line or beyond, Drug combination	Sym	2012-11-29	First full publication	1.21 (0.69-2.11)	-	1.2 (0.72-2.02)	-	No Benefit
Irinotecan	Pancreatic cancer, Neoadjuvant, 1st line, Drug combination	-	-	-	-	-	-	-	No RCT
Irinotecan	Pancreatic cancer, Advanced, 2nd line, 3rd line or beyond, Drug combination	-	-	-	-	-	-	-	No RCT
Lenalidomide	Multiple Myeloma, Post-auto, 1st line, Single agent	McCarthy (CALGB 100104)	2012-05-10	First full publication	0.52 (0.26-1.02)	-	0.37 (0.26-0.53)	-	PFS
		Attal (IFM2005-02)	2012-05-10	First full publication	1.06 (0.8-1.41)	0.80 (0.40-1.58)	0.5 (0.33-0.75)	0.42 (0.32-0.57)	PFS

© 2021 Herbrand AK et al. *JAMA Network Open.*

		Palumbo (RV-MM-PI-209)	2014-09-04	First full publication	0.72 (0.37-1.38)	0.80 (0.52-1.24)	0.5 (0.31-0.8)	0.44 (0.35-0.55)	PFS
		Holstein (CALGB 100104)	2017-09-01	Updated analysis	0.61 (0.46-0.8)	0.78 (0.52-1.18)	0.57 (0.46-0.71)	0.55 (0.46-0.65)	PFS
Lenalidomide	Multiple Myeloma, Post-auto, 1st line, Drug combination	Gay (RV-MM-EMN-441)	2015-11-17	First full publication	1.53 (0.79-2.98)	-	0.84 (0.59-1.2)	-	No Benefit
Lenalidomide	DLBCL, Palliative, 3rd line or beyond, Single agent	Czuczman, (DLC-001)	2014-12-06	Abstract	0.91 (0.59-1.41)	-	0.64 (0.41-0.99)	-	PFS
		Czuczman (DLC-001)	2017-08-01	First full publication	0.91 (0.6-1.39)	-	0.64 (0.42-0.98)	-	PFS
Lomustin	Glioblastoma, Advanced, 2nd line, 3rd line or beyond, Drug combination	Taal (BELOB Study)	2014-07-15	First full publication	-	-	-	-	No RCT
		Weathers (NA2)	2016-07-12	First full publication	-	-	0.71 (0.43-1.18)	-	No Benefit
		Wick (EORTC 26101)	2017-11-16	First full publication	0.95 (0.74-1.21)	-	0.49 (0.39-0.61)	0.55 (0.39-0.77)	PFS
Lomustin	Glioblastoma, Advanced, 2nd line, 3rd line or beyond, Single agent	-	-	-	-	-	-	-	No RCT
Lomustin	Brain Tumor WHO Grading III, Advanced, 2nd line, 3rd line or beyond, Single agent	-	-	-	-	-	-	-	No RCT

© 2021 Herbrand AK et al. *JAMA Network Open.*

Midostaurin	AML, Post-allo, 3rd line or beyond, Single agent	-	-	-	-	-	-	-	No RCT
Nab PACLITAXEL	Pancreatic cancer, Advanced, 2nd line, 3rd line or beyond, Drug combination	-	-	-	-	-	-	-	No RCT
Nivolumab	Melanoma, Adjuvant, 1st line, Single agent	Weber (CheckMate 238)	2017-09-10	First full publication	-	-	0.65 (0.51-0.83)	-	PFS
Nivolumab	Head and Neck, Advanced, 2nd line, Single agent	Gillison, (CheckMate 141)	2016-04-20	Abstract	0.7 (0.51-0.96)	-	-	-	OS
		Ferris, (CheckMate 141)	2016-10-08	First full publication	0.7 (0.51-0.96)	-	0.89 (0.7-1.13)	-	OS
		Ferris, (CheckMate 141)	2018-04-17	Updated analysis	0.68 (0.54-0.86)	-	0.87 (0.68-1.11)	-	OS
Nivolumab	Hodgkin Lymphoma, Palliative, 3rd line or beyond, Single agent	-	-	-	-	-	-	-	No RCT
Nivolumab	SCLC, Advanced, 2nd line, 3rd line or beyond, Single agent	-	-	-	-	-	-	-	No RCT
Nivolumab	Hodgkin Lymphoma, Palliative, 3rd line or beyond, Single agent	-	-	-	-	-	-	-	No RCT
		-	-	-	-	-	-	-	No RCT

© 2021 Herbrand AK et al. *JAMA Network Open.*

Nivolumab	HCC, Advanced, 2nd line, 3rd line or beyond, Single agent	-	-	-	-	-	-	-	No RCT
Novo-TTF	Glioblastoma, Advanced, 2nd line, Single agent	Stupp (EF-11)	2012-05-18	First full publication	0.86 (0.66-1.12)	-	0.81 (0.6-1.09)	-	No Benefit
		Kanner (EF-11)	2014-09-16	Updated analysis	0.69 (0.52-0.92)	-	-	-	OS
Novo-TTF	Glioblastoma, Advanced, 1st line, Drug combination	Stupp (EF-14)	2014-11-01	Abstract	0.75 (0.57-0.98)	-	0.63 (0.48-0.83)	-	OS
		Stupp (EF-14)	2015-12-15	First full publication	0.74 (0.56-0.98)	-	0.62 (0.43-0.89)	-	OS
		Stupp (EF-14)	2017-12-19	Updated analysis	0.63 (0.53-0.76)	-	0.63 (0.52-0.76)	-	OS
Obinutuzuma b	DLBCL, Induction, 2nd line, Drug combination	-	-	-	-	-	-	-	No RCT
Obinutuzuma b	CLL/SLL, Palliative, 3rd line or beyond, Drug combination	-	-	-	-	-	-	-	No RCT
Olaratumab	Soft Tissue Sarcoma, Advanced, 2nd line, Drug combination	Tap (NCT0118596 4)	2016-06-09	First full publication	0.46 (0.3-0.71)	-	0.67 (0.44-1.02)	-	OS
Osimertinib	NSCLC, Adenocarcinoma , Advanced, 1st line, Single agent	Ramalingam (FLAURA)	2017-09-09	Abstract	-	-	0.46 (0.37-0.57)	-	PFS
		Soria (FLAURA)	2017-11-18	First full publication	0.63 (0.45-0.88)	-	0.46 (0.37-0.57)	-	OS

© 2021 Herbrand AK et al. *JAMA Network Open.*

Osimertinib	NSCLC, Adenocarcinoma, Advanced, 2nd line, 3rd line or beyond, Single agent	Mok (AURA3)	2017-02-16	First full publication	-	-	0.3 (0.23-0.41)	-	PFS
Pembrolizumab	NSCLC, Adenocarcinoma, Advanced, 1st line, Single agent	Reck (KEYNOTE-024)	2016-10-09	First full publication	0.63 (0.47-0.86)	-	0.5 (0.37-0.68)	-	OS
		Lopes (KEYNOTE-042)	2018-06-07	First full publication	0.81 (0.71-0.93)	0.74 (0.59-0.94)	-	0.5 (0.37-0.68)	OS
Pembrolizumab	NSCLC, Adenocarcinoma, Advanced, 1st line, Drug combination	Langer (KEYNOTE-021)	2016-10-10	First full publication	0.9 (0.42-1.91)	-	0.53 (0.31-0.91)	-	PFS
		Gandi (KEYNOTE-189)	2018-04-16	First full publication	0.49 (0.38-0.64)	0.60 (0.34-1.04)	0.52 (0.43-0.64)	0.52 (0.43-0.63)	PFS
Pembrolizumab	NSCLC, Squamous, Advanced, 1st line, Single agent	Reck (KEYNOTE-024)	2016-10-09	First full publication	0.63 (0.47-0.86)	-	0.5 (0.37-0.68)	-	OS
		Lopes (KEYNOTE-042)	2018-06-07	First full publication	0.81 (0.71-0.93)	0.74 (0.59-0.94)	-	0.5 (0.37-0.68)	OS
Pembrolizumab	NSCLC, Squamous, Advanced, 1st line, Drug combination	Luis (KEYNOTE-407)	2018-05-20	Abstract	-	-	-	-	No Benefit
		Paz-Ares (KEYNOTE-407)	2018-09-25	First full publication	0.64 (0.49-0.85)	-	0.56 (0.45-0.7)	-	OS

© 2021 Herbrand AK et al. *JAMA Network Open.*

Pembrolizumab	Urothelial Cancer, Advanced, 2nd line, 3rd line or beyond, Single agent	Bellmunt (KEYNOTE-045)	2017-02-17	First full publication	0.73 (0.59-0.91)	-	0.98 (0.81-1.19)	-	OS
Pembrolizumab	Urothelial Cancer, Advanced, 1st line, Single agent	-	-	-	-	-	-	-	No RCT
Pembrolizumab	Colorectal Cancer, Advanced, 2nd line, 3rd line or beyond, Single agent	-	-	-	-	-	-	-	No RCT
Pembrolizumab	SCLC, Advanced, 2nd line, 3rd line or beyond, Single agent	-	-	-	-	-	-	-	No RCT
Pertuzumab	Breast Cancer, Neoadjuvant, 1st line, Drug combination	Gianni (NeoSphere)	2011-12-06	First full publication	-	-	-	-	No RCT
Ramucirumab	NSCLC, Adenocarcinoma, Advanced, 3rd line or beyond, Drug combination	Garon (REVEL)	2014-06-02	First full publication	0.86 (0.75-0.98)	-	0.76 (0.68-0.86)	-	OS
		Yoh (NCT01703091)	2016-07-18	First full publication	0.86 (0.56-1.32)	0.76 (0.76-0.98)	0.83 (0.59-1.16)	0.77 (0.69-0.86)	OS
Regorafenib	HCC, Advanced, 2nd line, Single agent	Bruix1, (RESORCE)	2016-06-30	Abstract	0.62 (0.5-0.78)	-	0.46 (0.37-0.56)	-	OS

© 2021 Herbrand AK et al. *JAMA Network Open.*

		Bruix (RESORCE)	2016-12-06	First full publication	0.63 (0.5-0.79)	-	0.46 (0.37-0.56)	-	OS
Rituximab	Marginal zone lymphoma, Induction, 1st line, Drug combination	Zucca (IELSG-19)	2013-02-10	First full publication	1.13 (0.52-2.45)	-	0.63 (0.39-1.02)	-	No Benefit
		Zucca (IELSG-19)	2017-06-10	Updated analysis	1.24 (0.69-2.23)	-	0.62 (0.42-0.92)	-	PFS
Rituximab	Follicular Lymphoma, Maintenance, 1st line, Single agent	Ghielmini (SAKK 35/98)	2004-02-19	First full publication	-	-	0.61 (0.4-0.93)	-	PFS
		Martinelli (SAKK 35/98)	2010-08-09	Updated analysis	0.63 (0.37-1.06)	-	0.59 (0.39-0.88)	-	PFS
Rituximab	Posttransplant lymphoproliferative disorder (PTLD), Induction, 1st line, Single agent	-	-	-	-	-	-	-	No RCT
Rituximab	Follicular Lymphoma, Induction, 1st line, Single agent	-	-	-	-	-	-	-	No RCT
Sorafenib	AML, Post-allo, 1st line, 2nd line, 3rd line or beyond, Single agent	-	-	-	-	-	-	-	No RCT
Sorafenib	AML, Post-allo, 1st line, 2nd line, 3rd line or beyond, Drug combination	-	-	-	-	-	-	-	No RCT
Temozolomide	Brain Tumor WHO Grading	Baumert (EORTC 22033-26033)	2015-05-20	Abstract	-	-	1.1 (0.6-1.9)	-	No Benefit

© 2021 Herbrand AK et al. *JAMA Network Open.*

	II, Advanced, 1st line, Single agent								
Temozolomide	Brain Tumor WHO Grading II, Advanced, 1st line, Single agent	Baumert (EORTC 22033-26033)	2016-09-27	First full publication	-	-	1.04 (0.56-1.93)	-	No Benefit
Temozolomide	Neuro-Endocrine Tumour, Advanced, 1st line, Drug combination	-	-	-	-	-	-	-	No RCT
Temozolomide	Neuro-Endocrine Tumour, Advanced, 2nd line, 3rd line or beyond, Drug combination	-	-	-	-	-	-	-	No RCT
Thiotepa	Primary central nervous system Lymphoma, Induction, 1st line, Drug combination	Ferreri (IELSG-32)	2015-06-20	Abstract	-	-	-	-	No Benefit
Thiotepa	Primary central nervous system Lymphoma, Induction, 1st line, Drug combination	Ferreri (IELSG-32)	2016-04-06	First full publication	0.78 (0.48-1.26)	-	0.72 (0.46-1.13)	-	No Benefit
Zoledronic acid	Breast Cancer, Adjuvant, 1st line, Drug combination	Brufsky (Z-FAST 2012)	2011-10-10	First full publication	-	-	0.87 (0.49-1.54)	-	No Benefit
		Coleman (ZO-FAST 2013)	2012-10-09	First full publication	0.69 (0.42-1.13)	-	0.66 (0.44-0.99)	0.72 (0.52-1.01)	OS

© 2021 Herbrand AK et al. *JAMA Network Open.*

		Coleman (AZURE 2014)	2014-07-15	First full publication	0.81 (0.63-1.04)	0.78 (0.63-0.98)	0.77 (0.61-0.97)	0.75 (0.62-0.91)	OS

Abbreviations: OLU = Off-label use; HR = Hazard ratio; OS = Overall Survival; CI = Confidence interval; PFS = Progression-free survival; RCT = Randomized-controlled trial; VTD = Bortezomib, Thalidomide and Dexamethasone; VRD = Bortezomib, Lenalidomide and Dexamethasone;

© 2021 Herbrand AK et al. *JAMA Network Open.*

eTable 3. Patient, Disease, and Treatment Characteristics of Reimbursement Requests and Their Associations With Reimbursement Decisions

	**Reimbursement Requests**		**Univariable**		**Multivariable Using Evidence OS**		**Multivariable Using Evidence OS or PFS**	
**Factor**	**Approval *(n = 295)***	**Disapproval *(n = 96)***	**OR (CI 95%)**	**P value**	**OR (CI 95%)**	**P value**	**OR (CI 95%)**	**P value**
Age, median (IQR)	64 (54, 71)	64 (54, 73)	0.99 (0.97, 1.01)	0.16	0.99 (0.97, 1.01)	0.31	0.99 (0.97, 1.01)	0.27
Sex – male, n (%)	178 (60)	52 (54)	1.28 (0.79, 2.08)	0.32	1.30 (0.77, 2.19)	0.32	1.26 (0.75, 2.12)	0.39
Tumor type – solid, n (%)	178 (60)	77 (80)	0.34 (0.19, 0.62)	< 0.001	0.61 (0.29, 1.24)	0.19	0.54 (0.26, 1.11)	0.09
Targeted therapy, n (%)	46 (16)	15 (16)	1.00 (0.52, 1.93)	1.00	0.56 (0.25, 1.24)	0.15	0.60 (0.27, 1.34)	0.21
Checkpoint inhibitor, n (%)	58 (20)	32 (33)	0.46 (0.26, 0.80)	0.006	0.56 (0.31, 1.02)	0.06	0.57 (0.31, 1.03)	0.06
Line of treatment, n (%)								
First Line	157 (53)	43 (45)	Reference	-	Reference	-	Reference	
Second line	88 (30)	35 (36)	0.66 (0.39, 1.14)	0.14	0.54 (0.30, 0.97)	0.04	0.70 (0.38, 1.30)	0.26
Third line of beyond	49 (17)	18 (19)	0.73 (0.38, 1.42)	0.36	0.58 (0.28, 1.19)	0.14	0.83 (0.39, 1.77)	0.62
Evidence for OS benefit	79 (27)	31 (32)	0.76 (0.45, 1.27)	0.30	0.85 (0.47, 1.54)	0.60	-	-
Evidence for OS or PFS benefit	133 (45)	35 (36)	1.45 (0.89, 2.36)	0.14	-	-	1.79 (1.01, 3.18)	0.05

Results from multivariable multilevel logistic regression analysis to investigate whether available evidence for OS benefit, evidence for OS or PFS benefit, patient, disease or treatment characteristics are associated with a higher chance for reimbursement. A higher odds ratio (OR) indicates higher chance for reimbursement by the health insurer.

© 2021 Herbrand AK et al. *JAMA Network Open.*

eTable 4. Sensitivity Analysis and Missing Imputation Calculations

**Model**	**Benefit**	**OR for reimbursement**	**95% CIs**
**Clustering Models**			
Multilevel logistic regression clustered by health insurer	OS benefit	0.76	0.46-1.27
	OS or PFS benefit	1.46	0.91-2.38
Multilevel logistic regression clustered by patient	OS benefit	0.73	0.42-1.27
	OS or PFS benefit	1.45	0.88-2.40
Logistic regression, no clustering	OS benefit	0.76	0.47-1.26
	OS or PFS benefit	1.44	0.91-2.33
**Trial model**			
Multilevel logistic regression, clustered by health insurer and patients	OS benefit	0.33	0.13 – 0.84
	OS of PFS benefit	0.95	0.36 – 2.47
**Multivariable, Multilevel logistic regression**			
Including all variable	OS benefit	0.85	(0.47, 1.54)
	OS of PFS benefit	1.79	(1.01, 3.18)
Without line of treatment as variable	OS benefit	1.02	0.58 – 1.79
	OS or PFS benefit	2.02	1.20 – 3.38
**Missing Imputations**			
Multilevel logistic regression	OS benefit	0.75	0.41 – 1.39
	OS or PFS benefit	1.40	0.82 – 2.37

Odds Ratio > 1 indicates higher chance of acceptance of reimbursement in the presence of exposure variable.

Abbreviations: OR – Odds Ratio; OS – Overall Survival; PFS – Progression-free Survival;

© 2021 Herbrand AK et al. *JAMA Network Open.*
